# The Golgi vesicle tether p115 can bind directly to the ER exit site organiser Sec16A

**DOI:** 10.1242/jcs.264894

**Published:** 2026-06-30

**Authors:** Igor Yakunin, Alison K. Gillingham, Conceição Pereira, David C. Gershlick, Sean Munro

**Affiliations:** 1 MRC Laboratory of Molecular Biology, Francis Crick Avenue, Cambridge CB2 0QH, UK; 2 Cambridge Institute for Medical Research, University of Cambridge, Cambridge CB2 0XY, UK

**Keywords:** ER exit site, Golgi, Membrane traffic, COPII vesicles

## Abstract

Newly made secretory and membrane proteins exit the endoplasmic reticulum (ER) in COPII vesicles that form at specialised ER exit sites. These exit sites are typically near the early Golgi compartments that receive these vesicles. A key player in the delivery of vesicles to the early Golgi is p115 (also known as USO1), a homodimer with a folded head domain and a coiled-coil tail that is anchored to Golgi membranes. p115 has been shown to capture vesicles and to bind to SNARE proteins to promote membrane fusion. Here, we report that the head domain of human p115 can bind directly to Sec16A, a large scaffolding protein that organises ER sites and promotes COPII vesicle formation. Structural prediction and deletion mapping defined the region of interaction to a conserved motif in the unstructured N-terminal region of Sec16A, and mutations in p115 that block motif binding reduced the efficiency of secretion. This interaction could potentially allow a subset of p115 molecules to reach across from the early Golgi to ER exit sites to contribute to the large-scale organisation of the early secretory pathway.

## Introduction

The Golgi apparatus is the major sorting hub in the secretory pathway. It receives newly made lipids and proteins from the endoplasmic reticulum (ER) and then, after they move through the cisternae of the Golgi stack, they are sorted to the cell surface or the compartments of the endocytic system (Glick and Luini, 2011; Pantazopoulou and Glick, 2019). Export from the ER is mediated by the COPII coat that forms vesicles and other carriers at ER exit sites (Downes and Zanetti, 2025; Gomez-Navarro and Miller, 2016; Peotter et al., 20s19). Soon after detaching from the ER, these carriers fuse to the ER–Golgi intermediate compartment (ERGIC) or the cis-Golgi, where the COPI coat forms vesicles that capture escaped ER residents to return them to the ER (Barlowe and Helenius, 2016). The ER exit sites and ERGIC are often closely opposed and there is growing evidence that they are organised as a functional unit that allows the rapid generation and consumption of the COPII and COPI vesicles that mediate anterograde and retrograde traffic, respectively (Downes et al., 2026; Farhan et al., 2025). In mammalian cells, there is a further level of organisation as there are ER exit sites throughout the ER network that fills the cytoplasm, whereas the Golgi apparatus is typically concentrated around the centrosome in the perinuclear region. In the cell periphery, the COPII vesicles fuse to the ERGIC, which then generates tubular carriers that move on microtubules toward the Golgi, or the ERGIC compartments themselves move *en bloc* to the Golgi once they reach a certain size (Stephens et al., 2000). Next to the Golgi in the centre of the cell, there is typically a concentration of ER exit sites, and the ERGIC that the COPII vesicles fuse with corresponds to the forming cis-Golgi compartment of the Golgi stack, which also receives the carriers from the peripheral ERGIC.

**Fig. 1:**
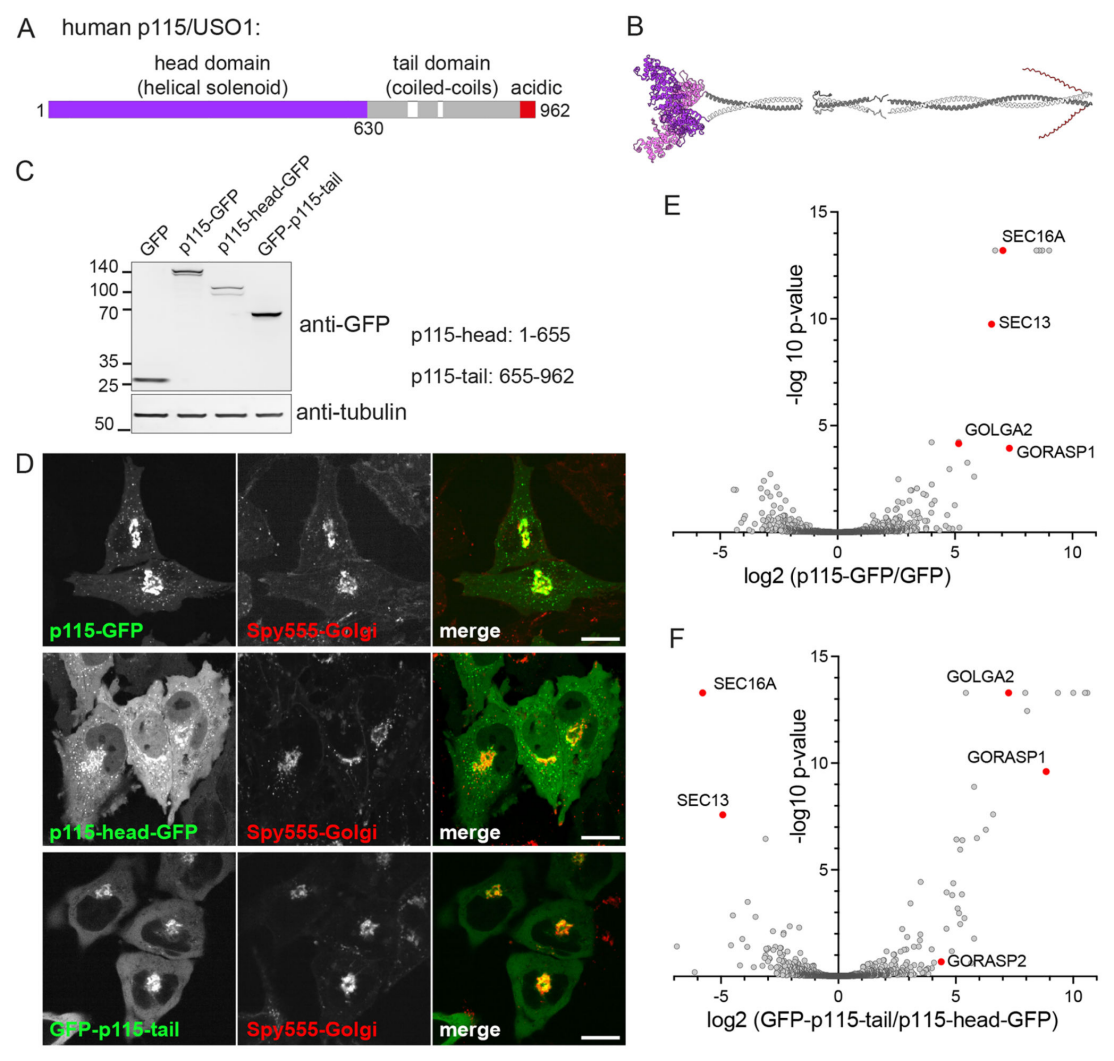
Identification of proteins co-precipitating with p115. (A) A schematic of p115: a globular head domain comprising armadillo repeats is followed by a C-terminal tail that forms a homodimeric coiled coil, which ends with a short highly acidic unstructured region that binds GM130. (B) Structural model generated using AlphaFold3 of a p115 dimer showing the head domain and predictions for the coiled-coil tail, which is shown in three parts with gaps at the regions predicted to be unstructured and hence likely to be flexible. Colouring is as in A. (C) Immunoblot showing expression in transfected HEK293T cells of GFP-tagged forms of p115 or its separate head and tail domains, with tubulin as a loading control. (D) Spinning disc confocal micrographs of live HeLa cells expressing the GFP-tagged forms of p115 shown in C and labelled with the Golgi vital stain SPY555-Golgi. All constructs label the Golgi, and the full-length and head domain forms also label the peripheral ER–Golgi intermediate compartment (ERGIC) structures. Images represent three independent experiments. Scale bars: 10 μm. (E) Volcano plot comparing enrichment of proteins co-precipitating with p115–GFP versus GFP from HEK293T cells. Values are means of biological triplicates. Highly enriched proteins involved in membrane traffic are indicated by red dots and named. The other highly enriched proteins are the centriolar components AKAP9 (AKAP450), CDK5RAP2, PCNT and PCM1, of which AKAP9 has been reported to be recruited to the Golgi by binding GM130 (Wu et al., 2016). For all protein identities, see Table S1. (F) Volcano plot comparing enrichment of proteins co-precipitating with GFP–p115-tail versus p115-head–GFP from HEK293T cells. Values are means of biological triplicates. The other highly enriched proteins with the p115 tail are the pericentriolar proteins noted in E. For all protein identities, see Table S1.

Several proteins have been proposed to contribute to the organisation of this ER–ERGIC unit of the secretory pathway, in addition to the SNARE proteins that drive the fusion of the vesicles with their destinations (Farhan et al., 2025). These include, in the ER, the peripheral scaffolding protein Sec16A, which helps recruit COPII coats into defined patches on the ER, and the integral membrane protein TANGO1 (also known as MIA3), which projects into both the ER lumen, where it is proposed to select specific cargo, and into the cytoplasm, where it is required to help organise the exit sites (Hughes and Stephens, 2010; Kurokawa and Nakano, 2019; McCaughey et al., 2021; Robinson et al., 2024). The cytosolic protein TFG can bind to COPII subunits and has been proposed to help in forming the COPII coat and also directing the clustering of COPII-coated carriers once they have budded (Hanna et al., 2017). On the ERGIC and cis-Golgi, various factors are likely to act very early in the transport process. The TRAPPIII complex activates Rab1, a small GTPase that has been proposed to recruit, among other effectors, the GBF1 exchange factor that activates Arf proteins to generate retrograde COPI vesicles (Galindo and Munro, 2023; Monetta et al., 2007). The golgin coiled-coil proteins GM130 (also known as GOLGA2) and GMAP210 (also known as TRIP11) are also on the ERGIC and cis-Golgi and contribute to efficient trafficking (Roboti et al., 2015; Seemann et al., 2000; Wong and Munro, 2014). One role of GM130 is to bind via its basic N-terminus to the acidic C-terminal region of the protein p115 (also known as USO1) (Nelson et al., 1998; Puthenveedu and Linstedt, 2004). The functional importance of this interaction is unclear as this part of p115 is not required for it to localise to the Golgi or to maintain Golgi structure and protein secretion. p115 was first identified in yeast as a temperature sensitive mutation, *Uso1-1*, that blocks ER-to-Golgi transport (Nakajima et al., 1991). Its mammalian ortholog was then discovered independently as a factor required in two *in vitro* assays for vesicular transport in the Golgi (Barroso et al., 1995; Sapperstein et al., 1995). It is localised to the ERGIC and the cis face of the Golgi, and, of the various putative structural proteins in this region of the pathway, it is the best conserved in evolution and appears to be essential in all organisms tested to date, ranging from mammals to yeast and protozoa (Kim et al., 2012; Pasquarelli et al., 2024; Seog et al., 1994). It has a head domain made up of armadillo repeats and a C-terminal coiled-coil domain that directs dimerization (Fig. 1A,B) (Sapperstein et al., 1995; Yamakawa et al., 1996).

Despite its clear importance in the early secretory pathway, the precise role and mechanism of action of p115 are not fully resolved. *In vitro* reconstitution of ER-to-Golgi transport in yeast showed that Uso1p acts to tether vesicles to Golgi membranes before the SNAREs act to drive fusion (Barlowe, 1997; Cao et al., 1998). This tethering step also requires the Rab1 ortholog Ypt1 and, in both yeast and mammals, this GTPase binds directly to p115 to promote its association with Golgi membranes, with Rab1 being proposed to bind to either the head domain or the coiled-coil region (Allan et al., 2000; An et al., 2009; Beard et al., 2005; Brandon et al., 2006). It has also been reported that the coiled-coil region of p115 interacts with SNAREs to promote their assembly, although a recent study in fungi suggests that the SNAREs interact with the head domain (Bravo-Plaza et al., 2023; Shorter et al., 2002). To further illuminate the role of p115, we sought new interaction partners for human p115 using a proteomic approach and found an unexpected interaction with the Sec16A component of ER exit sites.

## Results and Discussion

### Identification of interaction partners of p115

To illuminate the role of p115, we sought interaction partners by immunoprecipitation of GFP-tagged forms of the protein. Full-length p115 was expressed in HEK293T cells with GFP at its C-terminus, along with two truncations each comprising one of the two distinct parts of the protein: the head domain and the C-terminal coiled-coil. These three fusion proteins accumulated to similar levels and were detected, as expected, at the Golgi, with lower levels in the cytoplasm, indicating that they were correctly folded (Fig. 1C,D). Full-length p115-GFP was precipitated, with GFP alone used as a negative control, and associated proteins were identified with mass spectrometry (Fig. 1E). Comparison of p115–GFP to the GFP negative control revealed a strong enrichment for two known interaction partners: the Golgi coiled-coil protein GM130, which binds directly to the C-terminus of p115, along with GORASP1 (also known as GRASP65), which is known to bind directly to GM130 (Barr et al., 1998; Nakamura et al., 1997).

In addition to these known interactions, several proteins not previously reported to be associated with p115 were strongly enriched in the precipitations, including two known components of ER-to-Golgi traffic, Sec16A and Sec13 (Fig. 1E). Sec16A is a large protein that organises ER exit sites and promotes COPII vesicle budding by interaction with components of the Sar1:Sec23:Sec24 inner layer of the coat, and Sec13 is a β-propellor protein that forms a stable complex with Sec16A (Watson et al., 2006; Whittle and Schwartz, 2010). Sec13 also forms a complex with the COPII coat subunit Sec31 to form the outer layer of coat, but the latter protein was not enriched in the precipitates (Downes et al., 2026). To further investigate these interactions with p115, we compared the proteins that precipitated with the two halves of p115 and found that GM130 and its interactors precipitated with the C-terminal tail of p115 as expected, whereas Sec16A and Sec13 were the most strongly enriched interactors with the helical head domain (Fig. 1F).

### Mapping the region of Sec16A required for interaction with p115

**Fig. 2:**
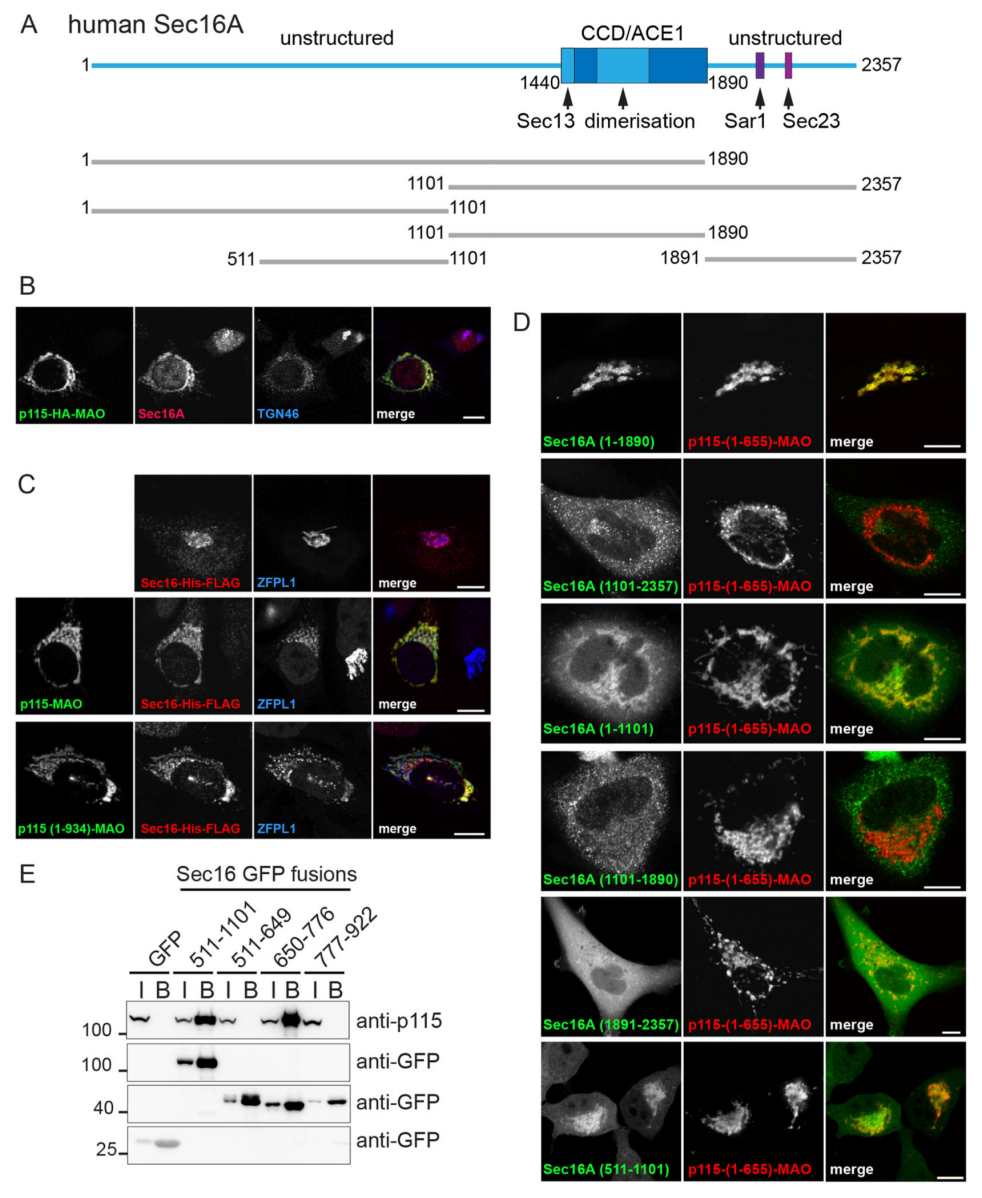
p115 interacts with Sec16A via part of the N-terminal unstructured region. (A) A cartoon schematic of human Sec16A showing the truncations used to map the p115-binding site. Most of the protein is predicted to be unstructured, apart from the central conserved domain [CCD, also referred to as the ancestral coatomer element 1 (ACE1)], which comprises a β-solenoid that forms a homodimer, along with a β-blade that binds the β-propellor protein Sec13 (Bhattacharyya and Glick, 2007; Whittle and Schwartz, 2010). The C-terminal unstructured region binds to Sec23 and its associated GTPase Sar1 (Bhattacharyya and Glick, 2007; Yorimitsu and Sato, 2012). (B) Confocal micrographs showing the capture of endogenous Sec16A by p115–HA–MAO on mitochondria in HeLa cells, with TGN46 (also known as TGOLN2) serving as a Golgi marker. (C) Confocal micrographs of HeLa cells expressing p115–HA–MAO and Sec16A–His6–FLAG, and labelled for the HA and FLAG tags along with the Golgi marker ZFPL1. (D) Confocal micrographs of HeLa cells expressing p115(1–655)–HA–MAO and the indicated fragments of Sec16A fused to GFP (as shown in A), and labelled for the HA tag. GFP is attached to the N-terminus of Sec16A, or at the C-terminus for Sec16A 511–1101. Micrographs in B–D represent three independent experiments. Scale bars: 10 μm. (E) Immunoblots showing interaction of endogenous p115 with the indicated GFP-tagged fragments of Sec16A, which were expressed in HEK293T cells and then precipitated. I, input; B, bound fraction. Blots represent three independent experiments.

Sec16A is predicted to be mostly unstructured with a central conserved domain (CCD) that forms a helical solenoid resembling part of a COPII subunit (Fig. 2A). This region of the protein homodimerises, and each monomer in the complex binds to the β-propellor protein Sec13, a protein present as a subunit in several other complexes (Dokudovskaya et al., 2011; Whittle and Schwartz, 2010). The C-terminal unstructured region of Sec16A binds to the Sec23 subunit of COPII and its associated Sar1 GTPase and has been reported to accelerate COPII coat formation (Kung et al., 2012; Yorimitsu and Sato, 2012). To determine where p115 binds to Sec16A, we expressed in cells a fusion of p115 to the C-terminal mitochondrial targeting signal from monoamine oxidase (MAO). Sec16A is normally located at ER exit sites, which are scattered throughout the cytoplasm with an accumulation next to the Golgi apparatus (Hughes et al., 2009; Watson et al., 2006). In the presence of mitochondrial p115, both endogenous and exogenous Sec16A relocated to the surface of mitochondria (Fig. 2B,C). The Golgi apparatus was fragmented by the presence of this mitochondrial p115, presumably due to the depletion of endogenous Sec16A and other p115-interacting proteins. Nonetheless, this *in vivo* relocation provided a means to map the part of Sec16A that interacts with p115, and so, a series of truncations of Sec16A were expressed along with a mitochondrial form of the p115 head domain (Fig. 2A,D). Residues 1–1101 of Sec16A are predicted to be primarily unstructured but were relocated by the mitochondrial p115 (Fig. 2D). In contrast, the rest of the protein, which contains the CCD and the Sec23-binding site, was not relocated when expressed either in its entirety or as fragments containing either the CCD or the Sec23-binding site (1101–1890 or 1891–2357; Fig. 2D). Further truncation of the N-terminal region showed that residues 511–1101 were still relocated by ectopic p115. To further map the region of binding, the region spanning residues 511–1101 was split into thirds and each fragment expressed as a GFP fusion and then precipitated from cells. Immunoblotting showed that residues 650–776 were sufficient for co-precipitation of p115 (Fig. 2E). Taken together, these results show that the head domain of p115 can interact with Sec16A via a 126-residue region in the N-terminal unstructured region of Sec16A.

### Structural prediction reveals the basis of a direct interaction between p115 and Sec16A

**Fig. 3:**
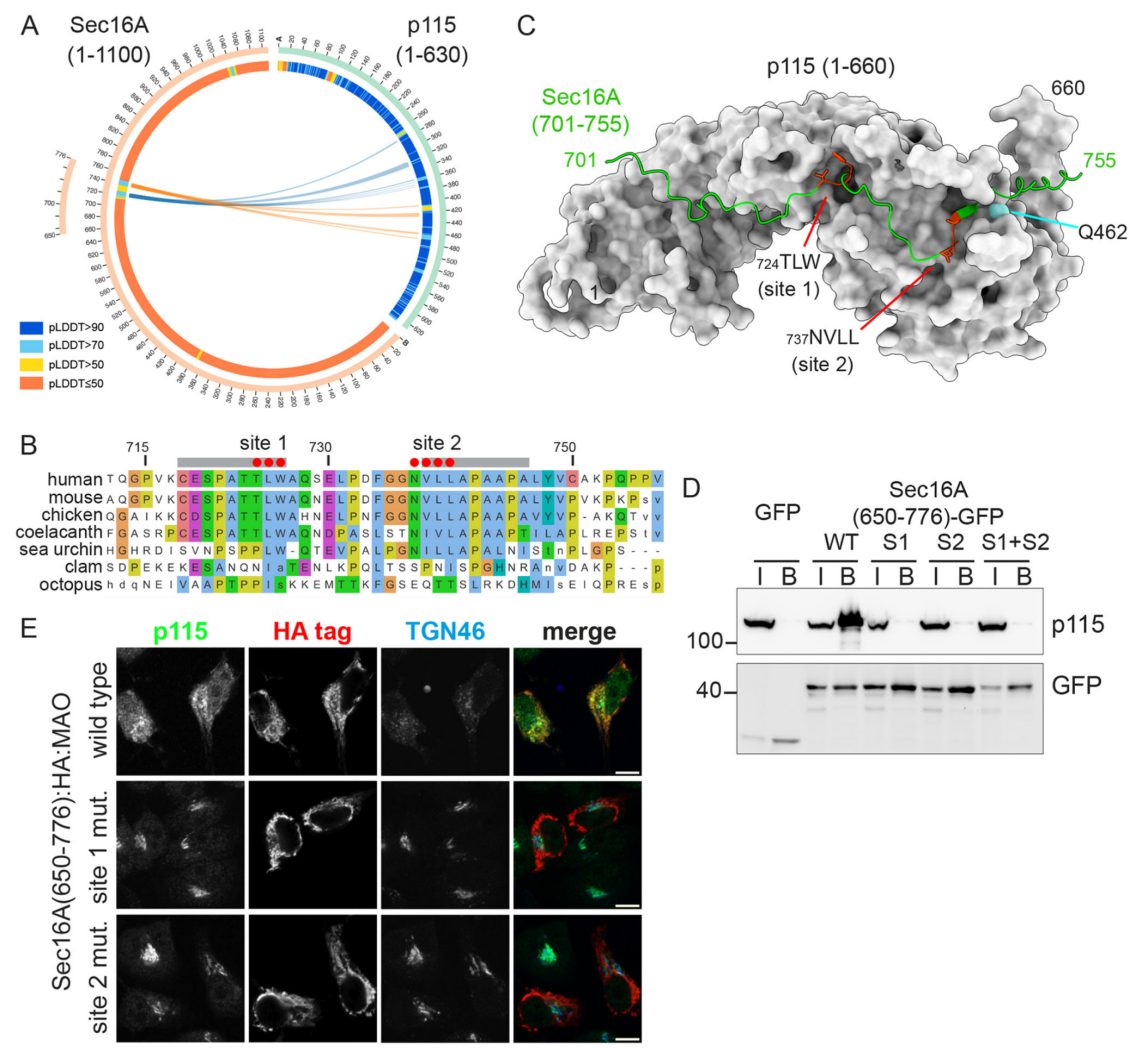
Prediction of a complex between Sec16A and the head domain of p115. (A) AlphaBridge visualisation of the output from an AlphaFold 3 prediction of a complex between the N-terminal region of Sec16A (1–1100) and the head domain of p115 (1–630). Shown in orange and blue are the two high-confidence interactions identified by AlphaBridge, both of which have a predicted interaction confidence score (piCS) of 0.91 (Álvarez-Salmoral et al., 2025 preprint). The predicted local distance difference test (pLDDT) score of ≤50 indicates that most of the indicated region of Sec16A is predicted to be unstructured. (B) ProViz alignment of human Sec16A (712–757) showing conservation in the indicated vertebrates and invertebrates. The two regions strongly predicted to interact with p115 are indicated in grey, along with the residues that were selected for mutation (red). These regions are well conserved in vertebrates but not in invertebrates. (C) AlphaFold 3 prediction of the complex formed between residues 701–755 of Sec16A and the head domain of p115. The residues of Sec16A lie along a groove in the surface of p115, with two regions predicted to interact with very high confidence. Shown in red are short stretches of residues in the middle of each region that were selected for mutation to alanine. (D) Immunoblots of anti-GFP precipitations from cells expressing the indicated forms of Sec16A (650–776) or GFP alone as a negative control, which were then probed for p115 and GFP. S1, site 1 mutations to alanine; S2, site 2 mutations to alanine. Blots represent three independent experiments. (E) Confocal micrographs of HeLa cells expressing the indicated mitochondrial forms of Sec16A (650–776) that were probed for endogenous p115, the Golgi marker TGN46 and the HA tag in the mitochondrial chimera. Images represent three independent experiments. Scale bars: 10 μm.

To obtain more insight into the interaction between p115 and the N-terminal region of Sec16A, we used the AlphaFold 3 structural prediction algorithm (Abramson et al., 2024). When the whole N-terminal region of Sec16A (residues 1–1100) was tested with the head domain of p115, two short regions of Sec16A gave a very confident prediction for binding (Fig. 3A). These two regions both lie within the 650–776 binding region mapped as described above (residues 718–726 and 738–746). The two regions are well conserved in vertebrates but do not appear to be conserved in invertebrates (Fig. 3B). In the predicted complex with p115, these regions are part of an unstructured stretch of residues that binds in a groove on the surface of the head domain, with this groove being on the opposite side to the attachment point for the coiled coil (Fig. 3C). To test this predicted structure, we examined the effect of mutating residues in Sec16A that appear to be involved in the interface with p115. When a short stretch of residues in either the first region (site 1) or the second region (site 2) was mutated to alanines (Fig. 3B), then region 650–776 lost the ability to co-precipitate endogenous p115 when expressed as a GFP fusion in transfected cells (Fig. 3D). When the region 650–776 of Sec16A was expressed on the surface of mitochondria, endogenous p115 relocated to the same organelle and, again, this activity was lost with either site 1 or site 2 mutations (Fig. 3E). These results indicate that a conserved bipartite motif in the N-terminal unstructured region of Sec16A can bind directly into a groove in the head domain of p115.

### Sec16A binding is required for full activity of p115 *in vivo*

**Fig. 4:**
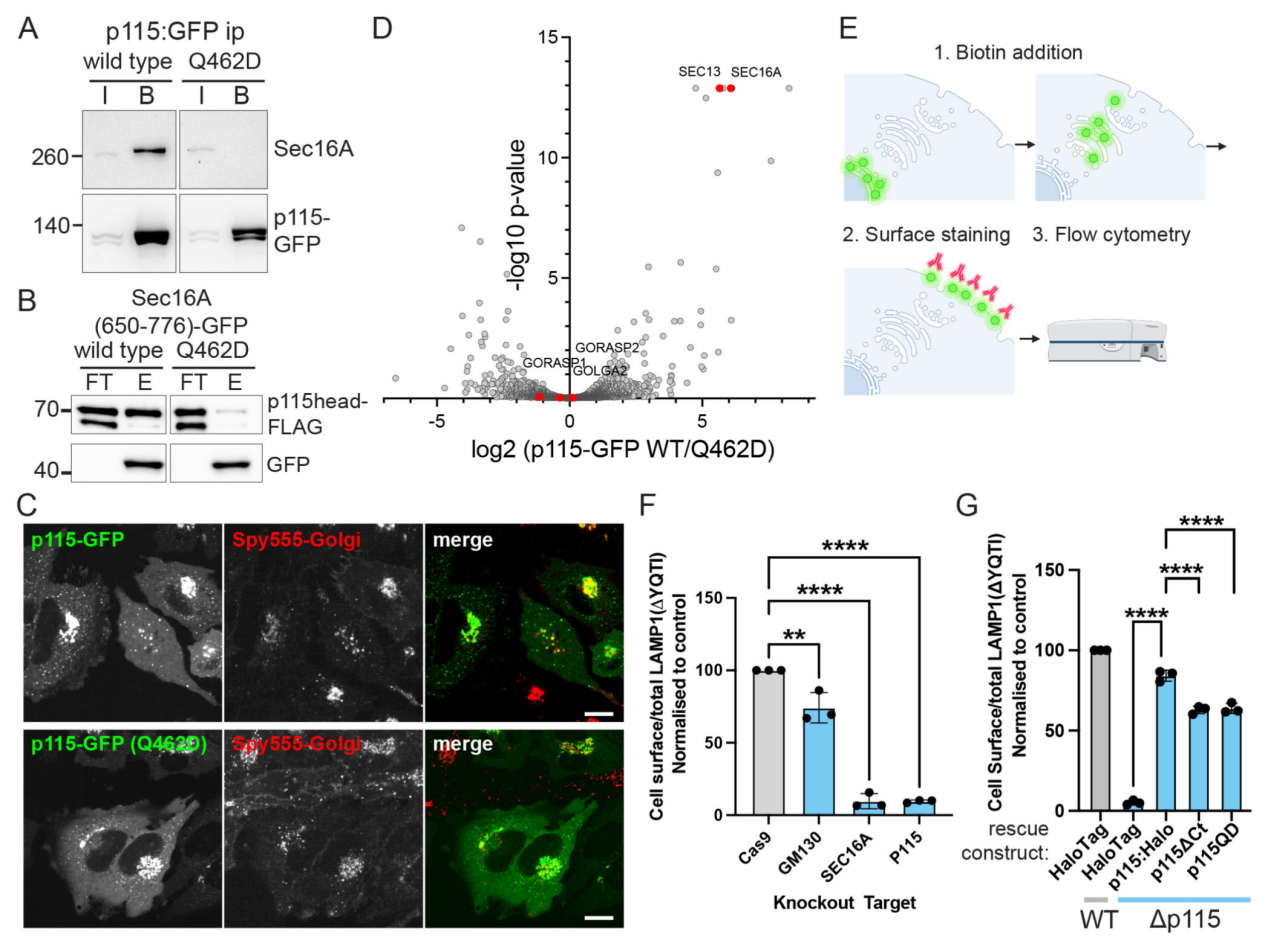
Mutation of the Sec16A-binding site in p115 reduces its activity. (A) Immunoblots showing binding between endogenous Sec16A and either wild-type full-length p115–GFP or full-length p115–GFP with the Q462D mutant, which were expressed in HEK293T cells and precipitated with anti-GFP. Blots were probed for Sec16A and GFP. I, input; B, bound fraction. Blots represent three independent experiments. (B) Immunoblots from a direct binding assay performed using FLAG-tagged p115 head domain (1–655) binding to Sec16A (650–776)–GFP on GFP-Trap beads. FT, flow-through/unbound fraction; E, eluate/bound fraction. Blots represent three independent experiments. (C) Confocal micrographs of live HeLa cells expressing full-length p115–GFP or the same with the Q462D mutation, and labelled with the Golgi stain SPY555-Golgi. Images represent three independent experiments. Scale bars: 10 μm. Volcano plot comparing enrichment of proteins co-precipitating with wild-type p115–GFP versus Q462D p115–GFP following transfection into HEK293T cells. Values are means of biological triplicates. Proteins found enriched with wild-type p115 are indicated, showing that the interaction with Sec16A and Sec13 are the ones that change with the mutant, whereas GM130 and its binding partners are unaffected. For all protein identities, see Table S1. (E) Schematic of the flow cytometry retention using selective hooks (RUSH) assay. Green circles represent LAMP1^ΔYQTI^–GFP cargo, red antibodies represent mCherry-tagged anti-GFP nanobodies. The LAMP1 reporter is held in the ER by a streptavidin–KDEL hook, which is released upon biotin addition, allowing LAMP1 to be transported to the cell surface, where it can be quantified by antibody binding and flow cytometry. (F) Bar graph showing the relative effects of GM130, p115 and Sec16A loss on anterograde trafficking (*n*=3). Grey, untreated Cas9 cells as control; blue, the same cells infected with lentivirus expressing CRISPR guides. Error bars indicate s.d. Statistical comparisons were made by one-way ANOVA followed by Tukey’s post hoc test. **P<0.01; ****P<0.0001. (G) Bar graph showing the degree of rescue of trafficking in cells lacking p115 by either wild-type or mutant p115. The first column represents a Cas9 cell line electroporated with HaloTag alone (wild-type), whereas all other columns represent the same cells treated with lentivirus expressing a CRISPR guide for p115 (Δp115) and expressed with HaloTag as a control or the indicated p115–HaloTag constructs (*n*=3). Statistical comparisons were performed as in F.

To determine the role of Sec16A binding in the activity of p115, we tested the effect of perturbing the groove in which Sec16A was predicted to bind. A glutamine at residue 462 lies against Sec16A in this binding grove (Fig. 3C). Mutation of this residue to aspartate (Q462D) resulted in loss of binding to Sec16A (Fig. 4A,B). When expressed in cells, the mutated p115 still localised to the Golgi and, when precipitated from cells, it was associated with similar levels of GM130 to those found with wild-type p115, indicating that the mutant form was not grossly misfolded (Fig. 4C,D). To test its activity *in vivo*, we used an assay for anterograde traffic: this involves releasing a GFP-tagged membrane protein from the ER that then travels to the cell surface, where it can be detected with a fluorescent antibody and, hence, its trafficking can be quantified by flow cytometry (Boncompain et al., 2012; Pereira et al., 2023) (Fig. 4E). The GFP-tagged reporter is a mutant form of LAMP1 (LAMP1^ΔYQTI^) that lacks an endocytosis signal, a change that results in it traveling from the ER to the Golgi and then directly to the cell surface where it accumulates (Chen et al., 2017; Ecard et al., 2024; Pereira et al., 2023). This LAMP1^ΔYQTI^ reporter has been previously used to successfully assay the role of a range of components of the conventional secretory pathway (Stalder et al., 2026). The *USO1* gene encoding p115 is essential for cell viability, but its expression could be transiently disrupted by delivery of CRISPR-Cas9 guides with lentivirus, followed by analysis of secretion 6 days later. As expected, this treatment blocked trafficking of the LAMP1^ΔYQTI^ reporter to the surface (Fig. 4F). To test the functional activity of p115, a guide-resistant cDNA was electroporated back into the CRISPR-Cas9-treated cells with a HaloTag on p115 to allow the transfected cells in the population to be identified with flow cytometry. Reintroduction of wild-type p115 showed good rescue of the p115 disruption, but this rescuing activity was reduced by the Q462D mutation (Fig. 4G). The reduction in rescuing activity was similar to that observed for p115 lacking the C-terminal region required for its ability to bind GM130 (p115ΔCT), an interaction that is well established but known to be not absolutely essential for its activity (Fig. 4F,G). Taken together, these results show that the Sec16A-binding site in p115 is functionally important for efficient membrane trafficking.

## Conclusions

In this paper, we show that the head domain of p115 can bind directly to a motif in the unstructured N-terminal region of Sec16A. This interaction does not appear to be responsible for the essential role of p115 in secretion in cultured cells. The essential role of p115 seems more likely to be that of promoting the assembly of SNARE complexes on the early Golgi, potentially directed by Rab1, although the details of these interactions remain to be resolved (Allan et al., 2000; An et al., 2009; Beard et al., 2005; Brandon et al., 2006). Indeed, the pattern of conservation of the sequence recognised by p115 suggests that this interaction arose early in vertebrate evolution as an extra function of p115 in addition to its function in vesicle tethering (Fig. 3B). Invertebrates and fungi only have a single gene encoding Sec16, but early in vertebrate evolution, this was duplicated to form Sec16A and Sec16B (Bhattacharyya and Glick, 2007; Budnik et al., 2011). Sec16B does not have the p115-binding motif described here, but its *in vivo* function is unclear as it is only expressed at low levels in a limited range of cell types and it is not essential for viability or fertility in mice, in contract to Sec16A, which is essential at a cellular level (Groza et al., 2023).

We speculate that p115 binding to Sec16A provides a link between ER exit sites and the ERGIC or cis-Golgi. Previous studies in yeast and plants have provided evidence for transient contacts between ER exit sites and ERGIC membranes that would provide the opportunity for efficient transfer of COPII vesicles between the two compartments (Glick, 2014; Kurokawa et al., 2014; Takagi et al., 2020). The C-terminus of p115 is known to bind the N-terminus of the golgin GM130, but the purpose of this is unclear as this part of p115 is not essential for its role in secretion (Nelson et al., 1998; Puthenveedu and Linstedt, 2004). The long regions of coiled coils in both proteins would allow the head domain of p115 to extend at least 200 nm from the ERGIC or cis-Golgi membrane where GM130 is anchored by its C-terminus. Binding Sec16A could enable a population of p115 molecules to hold the ER exit sites and ERGIC or cis-Golgi in close proximity. However, it is also possible that Sec16A binds to p115 molecules that are not membrane bound and, thus, could help attach the ER-derived COPII-coated vesicles to the tethers that will engage the destination membrane while they are still in the process of budding. Further work will be required to resolve these possibilities, but it seems likely that investigating these, and other interactions made by the machinery at ER exit sites, will reveal the mechanisms by which this complex but fundamentally important region of the cell is organised.

## Materials and Methods

### Cell culture and transfection

HeLa, HEK293T (American Type Culture Collection) and LAMP1^ΔYQTI^ RUSH HeLa cells (Pereira et al., 2023) were grown in Dulbecco’s modified Eagle medium (DMEM; Life Technologies) supplemented with 10% fetal bovine serum (FBS; Sigma-Aldrich) and penicillin-streptomycin at 37°C and 5% CO_2_. The cells were regularly checked for mycoplasma contamination (MycoAlert, Lonza Biosciences). For small-scale plasmid transfections, cells were split into six-well plates at a 1:6 dilution. After 24 h, cells were transfected with 1 μg of DNA, 4 μl of 1 mg/ml polyethylenimine (PEI; Polysciences) and 100 μl of Opti-MEM (Thermo Fisher Scientific). Either 12 or 24 h later, cells were plated onto slides for immunofluorescence or into chambered coverslips for live-cell imaging. For T75 flasks, 10 μg of DNA, 30 μl of PEI and 800 μl of Opti-MEM were used. For T175 flasks, 25 μg of DNA, 75 μl of PEI and 2 ml of Opti-MEM were used.

### Fluorescence microscopy

For immunofluorescence, cells were plated onto multi-well polytetrafluoroethylene-coated slides (Hendley-Essex) and placed in a humidified chamber inside a 37°C and 5% CO_2_ incubator for 12–24 h. Slide wells were washed with PBS, fixed for 20 min in 4% paraformaldehyde in PBS, rinsed three times with PBS, and permeabilised for 10 min in 0.5% Triton X-100 in PBS. After washing twice in PBS, cells were treated with blocking buffer (PBS, 20% FBS and 0.5% Tween 20) and incubated in blocking buffer containing primary antibodies for 1 h. The wells were washed thrice with PBS and incubated with secondary antibodies in blocking buffer for 1 h in the dark. After washing thrice with PBS, cells were mounted under a coverslip in VECTASHIELD with DAPI (Vectorlabs) and sealed with nail varnish. Images were obtained with on a Zeiss 780 confocal and processed using Fiji. Primary antibodies used were against Sec16A (ab70722, Abcam, RRID:AB_1270588, 1:100), TGN46 (AHP500G, Bio-Rad, RRID:AB_323104, 1:300), ZFPL1 (HPA014909, Sigma-Aldrich, RRID: AB_1859055, 1:300); p115 (ab184014, Abcam, 1:300), FLAG tag (F3165, Sigma-Aldrich, RRID:AB_259529, 1:500), HA tag (ROAHAHA, Sigma-Aldrich, RRID:AB_2687407) and His tag (ab18184, Abcam, RRID: AB_444306, 1:100), and were detected with species-specific Alexa Fluor-labelled secondary antibodies (A21202, A31572 and A21448, Invitrogen, 1:300). For live-cell imaging, cells were seeded into μ-slide chambered coverslips with ibiTreat coating (ibidi), in 300 μl of medium 24 h prior to imaging on a Zeiss 780 confocal microscope with the stage kept at 37°C and 5% CO_2_. Golgi in living cells were labelled with SPY555-Golgi (Spirochrome) in FluoroBrite DMEM (Thermo Fisher Scientific) supplemented with 10% FBS for 15 min at 37°C, then washed and imaged in the same medium on an Andor BC43 spinning disc confocal microscope.

### Precipitation of GFP-tagged proteins

One T175 flask of sub-confluent HEK293T cells was transfected with a plasmid expressing the GFP-tagged protein and, after 2 days, the cells were detached and pelleted by centrifugation. The pellet was resuspended in 500 μl lysis buffer [10 mM Tris-HCl pH 7.5, 150 mM NaCl, 0.5 mM EDTA, 0.5% Triton X-100, cOmplete protease inhibitors (Roche)] and incubated for 30 min at 4°C. The lysate was cleared at 16,000 ***g*** for 15 min, 25 μl was saved as an input control, and the rest was incubated at 4°C for 1 h with 50 μl GFP-Trap agarose bead slurry (Chromotek) that had been washed twice with 500 μl lysis buffer. At the end of the incubation, 25 μl of the supernatant was saved (unbound fraction) and the beads washed three times in wash buffer (10 mM Tris pH 7.5, 300 mM NaCl, 0.5 mM EDTA, cOmplete protease inhibitors). 60 μl of 2× sample buffer was added to the washed beads and, after heating at 100°C for 5 min, the beads were pelleted, the supernatant was transferred to a fresh tube and β-mercaptoethanol was added to a concentration of 10%. For mass spectrometry analysis, 30 μl of the sample was separated on a 12-well 4–20% tris-glycine gel (Thermo Fisher Scientific), stained with InstantBlue (Sigma-Aldrich) for 1 h, and each lane was cut into eight equal slices for trypsinisation and protein identification by label-free mass spectrometry. Analysis of samples by immunoblotting used primary antibodies against Sec16A (HPA005684, Sigma-Aldrich, RRID:AB_1079189, 1:1000), p115 (ab184014, Abcam, 1:2000), tubulin (YL1/2, sc-53029, Santa Cruz Biotechnology, RRID: AB_793541, 1:250), FLAG tag (F3165, Sigma-Aldrich, RRID:AB_259529, 1:1000), HA tag (3F10, Roche, RRID:AB_2314622, 1:1000), His tag (ab18184, Abcam, RRID:AB_444306, 1:1000) and GFP (SAB4301138, Sigma-Aldrich, RRID:AB_2750576, 1:2000). Blot transparency data are shown in Fig. S1.

### Protein identification by mass spectrometry

Gel slices were destained with 50% v/v acetonitrile and 50 mM ammonium bicarbonate, reduced with 10 mM dithiothreitol, and alkylated with 55 mM iodoacetamide. Digestion was performed with 6 ng/μl trypsin (Promega, UK) overnight at 37°C, and peptides extracted in 2% v/v formic acid and 2% v/v acetonitrile, and analysed by nano-scale capillary liquid chromatography (LC) coupled with tandem mass spectrometry (MS/MS) using an Ultimate U3000 HPLC (Thermo Scientific Dionex, San Jose, USA) to deliver a flow of approximately 300 nl/min. A μ-precolumn cartridge C18 Acclaim PepMap 100 (5 μm, 300 μm×5 mm; Thermo Scientific Dionex), trapped the peptides prior to separation on a C18 Acclaim PepMap100 (3 μm, 75 μm×250 mm; Thermo Scientific Dionex). Peptides were eluted with a 65 min gradient of acetonitrile (5 to 40%). The analytical column outlet was directly interfaced via a modified nano-flow electrospray ionisation source, with a hybrid linear quadrupole ion trap mass spectrometer (Orbitrap QExactive, Thermo Fisher Scientific). Data-dependent analysis was carried out, using a resolution of 60,000 for the full mass spectrum, followed by ten MS/MS spectra in the linear ion trap. Mass spectra were collected over a mass-to-charge ratio (*m/z*) range of 200–1800. MS/MS scans were collected using threshold energy of 35% Normalised Collision Energy for collision-induced dissociation. LC-MS/MS data were then searched against the UniProt database using the Mascot search engine programme (Matrix Science, UK). Database search parameters were set with a precursor tolerance of 10 ppm and a fragment ion mass tolerance of 0.8 Da. One missed enzyme cleavage was allowed and modifications for oxidized methionine, carbamidomethyl and phosphorylated serine, threonine and tyrosine were included. The mass spectrometry proteomics data have been deposited to the ProteomeXchange Consortium via the PRIDE (Perez-Riverol et al., 2019) partner repository with the dataset identifier PXD075617.

### Protein-binding assays

Two populations of HEK293T cells were used: one transfected with a plasmid expressing a GFP-tagged protein and the second expressing a FLAG-tagged protein. After 48 h, the cells were detached and lysed in 1 ml of lysis buffer as described above for GFP-tagged proteins. Lysates were incubated at 4°C for 1 h with either 100 μl of Anti-FLAG M2 Affinity Gel (Merck) used per T175 flask for FLAG-tagged protein or 40 μl of GFP-Trap beads for GFP-tagged proteins, with both sets washed twice in 500 μl lysis buffer prior to addition of lysates. The GFP-Trap beads underwent a two-step harsh wash (twice in 1% SDS in water; then thrice in 50 mM Tris pH 7.4, 1 M NaCl, 0.5 mM EDTA and 1% Triton X-100). Anti-FLAG beads were washed three times with lysis buffer, with the second wash supplemented with 1 M NaCl, and they were then incubated with 500 μl elution buffer (25 mM Tris-HCl pH 7.4, 250 mM NaCl, 1 mM EDTA, 100 μg/ml 3×FLAG peptide) for 15 min at room temperature. The eluted FLAG protein was incubated with the GFP-tagged protein on GFP-Trap beads for 4°C for 1 h. The beads were then washed three times with the original wash buffer (10 mM Tris pH7.5, 300 mM NaCl, 0.5 mM EDTA, cOmplete protease inhibitors) and eluted as for the GFP precipitation.

### RUSH secretion assay following lentiviral-based gene knockout

Oligonucleotide sequences corresponding to suitable guides were cloned into the pLVK lentiviral vector (Takara Bio) between two BbsI sites. 0.8×10^6^ Lenti-X 293T cells (Takara Bio) were seeded per well of a six-well plate coated with Matrigel (Corning). 125 μl Opti-MEM, 1 μg of each guide plasmid, 1 μg of Gag/Pol plasmid (Pereira et al., 2023), 0.34 μg of VSVG plasmid (Pereira et al., 2023) and 8.3 μl of P3000 reagent (Thermo Fisher Scientific) were combined with 125 μl of Opti-MEM containing 9.6 μl of Lipofectamine LTX (Thermo Fisher Scientific) and 2.4 μl of Lipofectamine 3000 (Thermo Fisher Scientific), incubated for 30 min and then added to the cells. Cell supernatants were collected after 3 days, passed through a 0.45 μm filter and stored at −80°C. For infection, 50 μl of a suspension of Cas9-expressing LAMP1^ΔYQTI^ HeLa cells (5×10^5^ cells/ml) were transduced with 200 μl of lentivirus suspension and centrifuged at 700 ***g*** at 37°C for 1 h in a 48-well plate. After 48 h, cells were transferred from the 48-well plate to a six-well plate. For rescue experiments, infections were scaled up accordingly. The p115 rescue constructs were engineered to contain silent mutations to prevent recognition by the p115 CRISPR guides. 6 days post infection, cells were trypsinised, placed in DMEM, washed three times with 3 ml Opti-MEM by centrifugation, and resuspended in 200 μl Opti-MEM. 10^6^ cells in 200 μl of Opti-MEM were added to 0.5 μg of DNA and electroporated with the Gene Pulser Xcell electroporator (Bio-Rad) using the default HeLa settings. Cells resuspended in 2 ml DMEM were transferred to six-well plates, 1 ml of DMEM containing HaloTag-646 ligand (Janelia) was added to a final concentration of 20 nM, and the cells were incubated overnight.

For the retention using selective hooks (RUSH) assay, 1 week after infection, cells were trypsinised, suspended in 5 ml DMEM, and centrifuged for 5 min at 500 ***g***. The cells were resuspended in 1 ml CO_2_-free medium (DMEM containing 25 mM HEPES pH 7.4), split equally between two 1.5 ml tubes, and 0.5 ml of CO_2_-free medium was added to one (labelled *t*=0). All tubes were placed in a 37°C hot block, 1 ml CO_2_-free medium with 1 mM biotin was added to the tubes apart from the one labelled *t*=0, and RUSH was allowed to progress for 35 min. Cells were centrifuged, resuspended in 100 μl of PBS containing 400 μg/ml mCherry anti-GFP nanobody (Addgene #109421) for 1 h, then washed twice in 0.5 ml PBS, resuspended in 0.4 ml PBS, and passed through a 40 μm cell strainer for flow cytometry (LSRFortessa, BD Biosciences). Data from three repeats were processed using FlowJo, and ANOVA testing used to compare the differences in ER exit site colocalisation in different cell populations (GraphPad Prism 9).

### Computational tools and methods

For mass spectrometric data, the DEP R package (v1.30.0) was used to analyse the label-free quantification (LFQ) intensities of each protein identified across the samples (Zhang et al., 2018). The data were filtered to exclude contaminants and reverse sequences. Any duplicated proteins were removed. Each sample was grouped with the two other repeats for each condition. A filter was applied with the rule that a protein had to be present across all three repeats of a condition to be included. The data were normalised and missing values were added by imputation. We opted to use a left-shifted Gaussian distribution with a downshift of 1.8 standard deviations and width of 0.3 standard deviations relative to the parent population. This generates a small and more tightly spaced distribution of normalised LFQ intensities. Missing values were then randomly picked from the distribution to allow calculation of the enrichment values and these were compared for each protein between samples to derive a *P*-value. Protein alignments were performed using ProViz, and structural prediction was performed using AlphaFold 3, followed by analysis in AlphaBridge and visualisation in ChimeraX (Abramson et al., 2024; Álvarez-Salmoral et al., 2025 preprint; Jehl et al., 2016; Pettersen et al., 2020).

## Supplementary Material

Table S1

Supplementary Figures

## Data Availability

The mass spectrometry proteomics data have been deposited to the ProteomeXchange Consortium via the PRIDE (Perez-Riverol et al., 2019) partner repository with the dataset identifier PXD075617. Plasmids are available from the authors upon reasonable request. All other relevant data and details of resources can be found within the article and its supplementary information.
