# Supplementary material for: The Golgi vesicle tether p115 can bind directly to the ER exit site organiser Sec16A
Source: J Cell Sci. Author manuscript; Available in PMC 2026 Aug 3. (PMC7619307; doi:10.1242/jcs.264894)
Supplement: Supplementary Figures [file EMS217163-supplement-Supplementary_Figures.pdf]

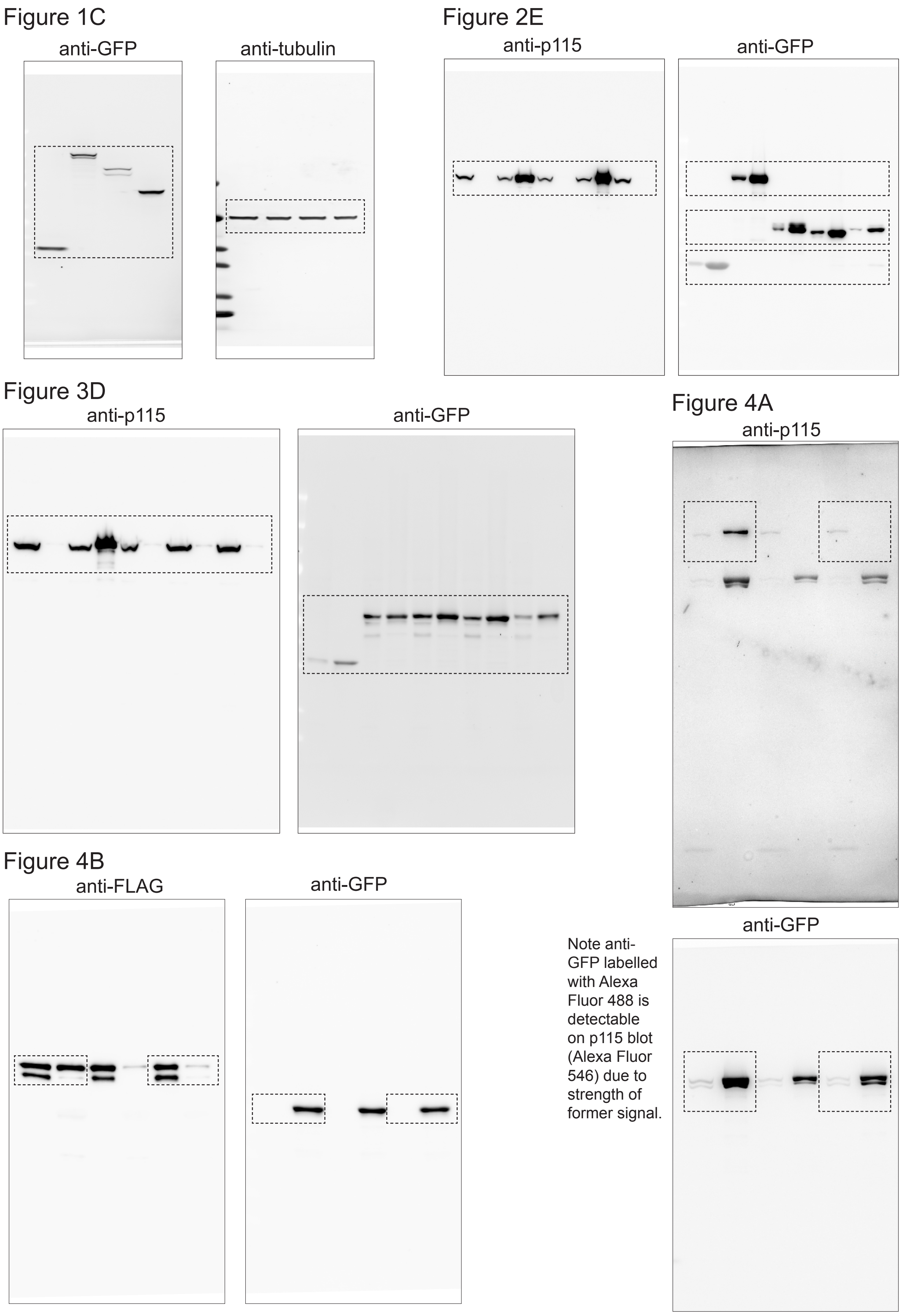

Fig. S1. Uncropped immunoblot images.

Journal of Cell Science • Supplementary information

Table S1. Mass spectrometry data shown in volcano plots

Available for download at
https://journals.biologists.com/jcs/article-lookup/doi/10.1242/jcs.264894#supplementary-data

Journal of Cell Science • Supplementary information
